# Infection‐Related Hospitalization and Incident Heart Failure: The Atherosclerosis Risk in Communities Study

**DOI:** 10.1161/JAHA.123.033877

**Published:** 2025-01-30

**Authors:** Rebecca L. Molinsky, Amil Shah, Melana Yuzefpolskaya, Bing Yu, Jeffrey R. Misialek, Bruno Bohn, David Vock, Richard MacLehose, Barry A. Borlaug, Paolo C. Colombo, Chiadi E. Ndumele, Junichi Ishigami, Kunihiro Matsushita, Pamela L. Lutsey, Ryan T. Demmer

**Affiliations:** ^1^ Division of Epidemiology and Community Health, School of Public Health University of Minnesota Minneapolis MN USA; ^2^ Cardiovascular Imaging Program, Departments of Medicine and Radiology Brigham and Women’s Hospital, Harvard Medical School Boston MA USA; ^3^ Division of Cardiology, Department of Medicine Columbia University Irving Medical Center New York NY USA; ^4^ Department of Epidemiology, Human Genetics and Environmental Sciences, School of Public Health University of Texas Health Science Center at Houston Houston TX USA; ^5^ Division of Biostatistics, School of Public Health University of Minnesota Minneapolis MN USA; ^6^ Department of Cardiovascular Medicine Mayo Clinic College of Medicine and Science Rochester MN USA; ^7^ Johns Hopkins Ciccarone Center for the Prevention of Heart Disease Johns Hopkins University School of Medicine Baltimore MD USA; ^8^ Department of Epidemiology and the Welch Center for Prevention, Epidemiology, and Clinical Research Johns Hopkins Bloomberg School of Public Health Baltimore MD USA; ^9^ Department of Epidemiology, Bloomberg School of Public Health Johns Hopkins University Baltimore MD USA; ^10^ Welch Center for Prevention, Epidemiology, and Clinical Research Johns Hopkins University Baltimore MD USA; ^11^ Division of Epidemiology, Department of Quantitative Health Sciences Mayo Clinic College of Medicine and Science Rochester MN USA

**Keywords:** epidemiology, heart failure, infections, Heart Failure, Epidemiology

## Abstract

**Background:**

The immune response to infections may become dysregulated and promote myocardial damage contributing to heart failure (HF). We examined the relationship between infection‐related hospitalization (IRH) and HF, HF with preserved ejection fraction, and HF with reduced ejection fraction.

**Methods and Results:**

We studied 14 468 adults aged 45 to 64 years in the ARIC (Atherosclerosis Risk in Communities) Study who were HF free at visit 1 (1987–1989). IRH was identified using select *International Classification of Diseases* (*ICD*) codes in hospital discharge records and was treated as a time‐varying exposure. HF incidence was defined as the first occurrence of either a hospitalization that included an *ICD, Ninth Revision* (*ICD‐9*) discharge code of 428 (428.0–428.9) among the primary or secondary diagnoses or a death certificate with an *ICD‐9* code of 428 or an *ICD, Tenth Revision* (*ICD‐10*) code of I50 among any of the listed diagnoses or underlying causes of death. We used multivariable‐adjusted Cox proportional hazards models to assess the association between IRH and incident HF, HF with reduced ejection fraction, and HF with preserved ejection fraction. Median follow‐up time was 27 years, 55% were women, 26% were Black, mean age at baseline was 54±6 years, 46% had an IRH, and 3565 had incident HF. Hazard ratio (HR) for incident HF events among participants who had an IRH compared with those who did not was 2.35 (95% CI, 2.19–2.52). This relationship was consistent across different types of infections. Additionally, IRH was associated with both HF with reduced ejection fraction and HF with preserved ejection fraction: 1.77 (95% CI, 1.35–2.32) and 2.97 (95% CI, 2.36–3.75), respectively.

**Conclusions:**

IRH was associated with incident HF, HF with reduced ejection fraction, and HF with preserved ejection fraction. IRH might represent a modifiable risk factor for HF pathophysiology.

**Nonstandard Abbreviations and Acronyms**
ARIC: Atherosclerosis Risk in Communities
HFpEF: heart failure with preserved ejection fraction
HFrEF: heart failure with reduced ejection fraction
IRH: infection‐related hospitalization

**Research Perspective**

**What Is New?**

- We found that several different types of hospitalized infections predicted increased risk for subsequent heart failure development during >20 years of follow‐up.
- Our findings were consistent for both heart failure with reduced and preserved ejection fraction.

**What Question Should Be Addressed Next?**

- It remains unknown whether a history of severe infection adds additional prognostic information above and beyond existing heart failure prediction scores.
- Future research to better understand the mechanisms potentially linking infections to heart failure pathophysiology are warranted.

An estimated >37 million individuals have heart failure (HF) globally,^1^ and prognosis after HF diagnosis is poor, with a <50% survival rate at 5 years,^2^, ^3^, ^4^ The total number of patients with HF continues to rise due to the growing aging population. The prognosis among patients with HF remains poor, and quality of life is severely reduced. A recent American Heart Association Presidential Advisory emphasized that the current pipeline for development of novel therapies is flat, necessitating innovative solutions to counteract increasing rates of cardiovascular death.^4^, ^5^

Left ventricular ejection fraction (LVEF) is generally viewed as a clinically useful phenotypic marker, commonly dichotomizing patients with HF into HF with reduced ejection fraction (HFrEF; LVEF<50%) or preserved ejection fraction (HFpEF; LVEF≥50%). Although HFrEF and HFpEF represent distinct clinical entities with respect to the causes and response to therapies, they share some common pathophysiologic pathways.^6^, ^7^

Inflammation plays a key role in the development of HFrEF and HFpEF^8^, ^9^, ^10^, ^11^ and elevated concentrations of proinflammatory biomarkers are common in both HFpEF and HFrEF.^12^, ^13^, ^14^ Although the drivers of inflammation are not fully understood, potential upstream triggers of the proinflammatory phenotype commonly characterizing HF are likely multifactorial. Infections represent 1 possible trigger of a proinflammatory phenotype. Several possibly mechanisms have been discussed linking infections to chronic inflammation including maladaptive innate immune system training,^15^ bacteremia and subsequent methylation of cardiac tissue,^15^ or the induction of endothelial dysfunction.^16^

Infections have been reported to both worsen symptoms in existing HF and to be a precipitant factor for acute HF.^17^, ^18^ Sepsis and pneumonia in particular have been linked to myocardial injury and depressed cardiac function leading to cardiac dysfunction.^18^, ^19^ More recently, the COVID‐19 has emerged as a possible contributor to HF.^18^, ^20^, ^21^, ^22^

Nevertheless, few studies have evaluated a broad range of infections in relation to incident HF and HF subtypes, and no study had explored this relationship in a large prospective cohort study with long‐term follow up.^23^, ^24^ Additionally, a history of severe infection is not currently considered in HF risk stratification.

The aim of our current study was to investigate the association between infection‐related hospitalizations (IRH) as a marker of severe infection, and incident HF during 31 years of longitudinal follow‐up in the ARIC (Atherosclerosis Risk in Communities) Study. We hypothesize that IRH will be associated with increased risk of incident HFrEF and HFpEF after accounting for a robust set of cardiometabolic risk factors.

## METHODS

### Data Availability

Because of the sensitive nature of the data collected for this study, requests to access the data set from qualified researchers trained in human subjects confidentiality protocols may be sent to the corresponding author.

### Study Population

The ARIC study is a prospective population‐based study of cardiovascular disease in adults aged 45 to 64 years who were recruited from 4 US communities between 1987 and 1989 (visit 1).^25^ Participants have attended additional follow‐up clinic visits and received phone calls (annually until 2012; twice yearly thereafter). The study protocol was approved by the institutional review boards of all participating centers, and all participants provided written informed consent at each clinic visit. In addition, the study has had continual institutional review board oversight. For the present primary analyses, we used longitudinal data from the visit 1 baseline until the end of 2018 (or December 31, 2017, among participants from the Jackson site). For our secondary analysis looking at HF subtypes, we used longitudinal data from 2005 until the end of 2018 (or December 31, 2017, among participants from the Jackson site), using covariates measures at visit 4 (1996–1998).

**Figure 1:**
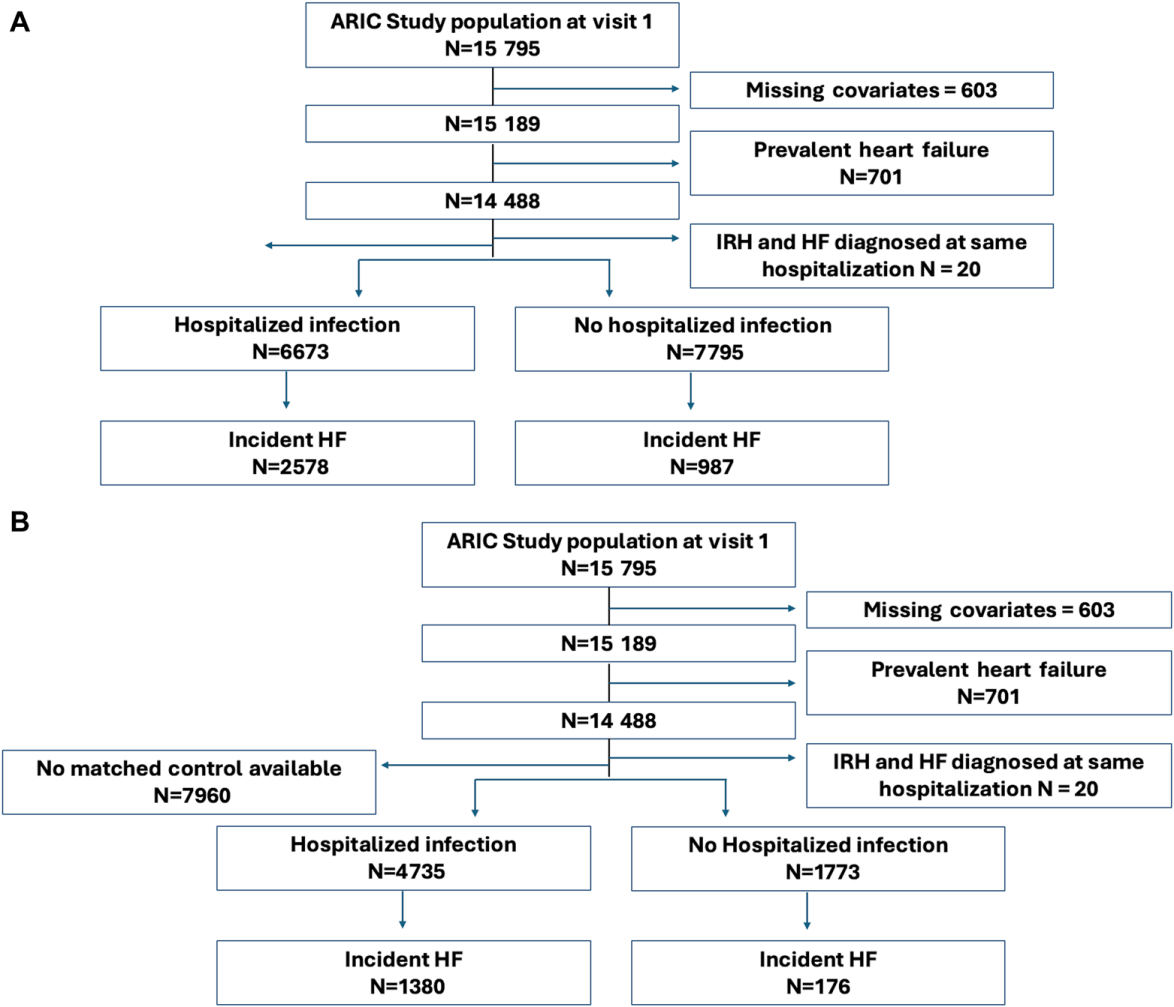
Study population flow chart for the full unmatched analysis (A) and the matched analysis (B). ARIC indicates Atherosclerosis Risk in Communities; HF, heart failure; and IRH, infection related hospitalization.

Of the 15 792 participants who attended visit 1, we excluded those with missing covariables information, race other than Black participants in the Minneapolis and Washington County center, and those who had prevalent HF. The same exclusions were applied to the HF subtype analysis, in which those with prevalent HF before 2005 were excluded (Figure 1).

### Heart Failure Definitions

Prevalent HF at baseline was defined as (1) an affirmative response to “Were any of the medications you took during the past 2 weeks for heart failure?” or (2) stage 3 or “manifest HF” by Gothenburg criteria.^26^, ^27^ All current medications (taken within the past 2 weeks) were brought into the clinic and documented. Incident HF events through December 31, 2018 (or December 31, 2017, among participants from the Jackson site) were identified through (1) annual telephone calls to ARIC cohort participants to identify all hospitalizations, (2) review of local hospital discharge indexes, and (3) retrieval of death certificates. HF incidence was defined as the first occurrence of either a hospitalization that included an *International Classification of Diseases, Ninth Revision* (*ICD‐9*) discharge code of 428 (428.0–428.9) among the primary or secondary diagnoses or else a death certificate with an *ICD‐9* code of 428 or an *International Classification of Diseases, Tenth Revision* (*ICD‐10*) code of I50 among any of the listed diagnoses or underlying causes of death.^27^ Validation of HF hospitalizations indicated that the positive predictive value of 428.x was 93% for acute decompensated HF and 97% for chronic HF.^28^

Starting in 2005, ARIC implemented an adjudication committee of HF hospitalizations based on chart abstraction as previously described.^29^ Briefly, hospitalizations or deaths with potentially HF‐related *ICD* codes were identified, and hospitalization records were abstracted and adjudicated by the ARIC HF Committee. Abstraction included results of imaging studies and LVEF when available. Participants with quantifiable LVEF were categorized as HFpEF (LVEF ≥50%) or HFrEF (LVEF<50%). If adjudication of the HF type was not possible, participants were defined as having “unknown” HF in all analyses.

### Infection‐Related Hospitalization Definitions

In ARIC, all hospitalization events were identified through phone calls, surveillance of local hospitals, and death interviews with proxies. The primary exposure variable is defined as the first occurrence of IRH, identified by selected *ICD‐9* or *ICD‐10* codes (Table S1) in the first 5 diagnostic positions, as previously done in ARIC.^30^ For the primary analysis, IRH was treated as a time‐varying exposure, with participants considered unexposed until their first IRH, after which they were considered exposed. A secondary analysis considered specific types of IRH (eg, respiratory, influenza, urinary tract, digestive tract, skin, blood/circulatory system, hospital‐acquired, and other infections).

### Risk Factors

Covariates were measured at baseline (visit 1) via questionnaires, clinical exam, and laboratory analysis of blood samples. These measures included age, sex, race (Black or White and included as a proxy for social, not biological, risk factors^31^), and education level (less than a high school degree, high school, general education diploma, or vocational school and at least some college). Participants brought bottles for medications and supplements taken in the prior 2 weeks to the clinic visit; medication names were recorded. In addition, physical activity was assessed at visit 1 via a modified Baecke questionnaire. A physical activity index score (1: lowest activity and 5: highest activity) was calculated based on intensity and time dedicated to sport and exercise. Smoking status (never, former, or current), and access to health care variables were collected, including insurance status (private insurance, Medicare/Medicaid only, none).

During the visit 1 clinical examination, fasting blood was collected for the assessment of lipid profile (total cholesterol, high‐density lipoprotein and low‐density lipoprotein). Diabetes was defined as a self‐reported physician diagnosis of diabetes, fasting glucose ≥126 mg/dL, ≥200 mg/dL if nonfasting, or reported pharmacological treatment for diabetes. In addition, body mass index was calculated as measured weight in kilograms divided by height in meters squared. Blood pressure was measured 3 times after a 5‐minute rest. The average of the last 2 blood pressure measurements was used for analysis. Use of antihypertensive medication was assessed by medications that participants brought to the clinic. Baseline prevalence of chronic kidney disease was identified by estimated glomerular filtration rate, atrial fibrillation was identified by ECGs, and coronary artery disease and stroke were defined via self‐report.

### Statistical Analysis

Baseline characteristics by exposure status were described using mean±SD for continuous variables and count (%) for categorical variables. Cox proportional hazards models were used to assess the relationship between IRH and incident HF. For our primary analysis, IRH was treated as time varying and defined using prespecified *ICD‐9* or *ICD‐10* codes (Table S1) in the first 5 positions of discharge diagnosis. A matched approach was also carried out to minimize potential confounding using the greedy method.^32^ Participants were individually 1:1 matched on baseline age (±1 year of index participant), sex, race/center, and diabetes status. In our matching method, a participant with infection (index) is matched at the time of infection to a participant without infection. The person‐time of the matched participant without infection begins on the same date as the index infection and contributes uninfected person‐time until incident HF, death, or December 31, 2018, or until they become infected; if they become infected, they then start contributing infected person‐time until censoring as described. Thus, they contribute both infected and uninfected person‐years to the analysis (emulating an as‐treated approach). A multivariable Cox regression was carried out with the following models: model 1: crude (matched sample); model 2: model 1+adjusted for covariates measured in 1987 to 1989: education, insurance, body mass index, smoking status, low‐density lipoprotein cholesterol, physical activity, hypertension medication, prevalent coronary artery disease, and systolic blood pressure.

In addition, an unmatched analysis was also carried out in which follow‐up time began at visit 1 (1987–1989) and accrued until date of HF diagnosis, loss to follow‐up, death, or December 31, 2018, whichever occurred first. Hazard ratios (HRs) and 95% CIs were reported. Multivariable models adjusted for the following variables: Model 1: age, sex, race/center education, health insurance; Model 2: model 1+physical activity, smoking status, body mass index; Model 3: model 2+diabetes, systolic blood pressure, antihypertensive medication use, low‐density lipoproteincholesterol, and prevalent coronary artery disease. Our secondary analyses explored specific IRH (respiratory, influenza, urinary tract, digestive tract, skin, blood/circulatory system, hospital‐acquired, and other infections).

We replicated these analyses with incident HFrEF and HFpEF as outcomes. Follow‐up time started in 2005 for these analyses, when HF adjudication began, to the occurrence of incident HFrEF or HFpEF, loss to follow‐up, death, or December 31, 2018, whichever occurred first. Participants who developed HF or were censored before 2005 were excluded from this analysis.

A sensitivity analysis defining IRH as a primary diagnosis (*ICD* diagnostic position 1) was conducted to examine the impact of *ICD* diagnostic position on the results. The proportional hazards assumption could not be directly tested because both the test for proportionality and our exposure are time varying. However, to empirically assess for meaningful violations of the proportional hazards assumption, we assessed the strength of observed associations in sensitivity analyses that restricted the maximum length of follow‐up time at 2.5 years (25th percentile), 6 years (50th percentile), or 12.3 years (75th percentile) to determine whether the relationship became notably weaker and lost clinical significance over time.

## RESULTS

Among 14 468 participants, median follow‐up time was 27 years, 55% were women, 26% were Black, and the mean age at baseline was 54±6 years (range: 44–66 years). Overall, 6673 participants (46%) had at least 1 IRH throughout the entire study duration, not limited to those who had an infection after an HF event. Baseline characteristics of patients stratified by the IRH status at the end of follow‐up both the unmatched and matched sample are presented in Table 1. When compared with patients with no IRH, patients with at least 1 IRH had higher mean body mass index, systolic blood pressure, total cholesterol, and greater prevalence of diabetes.

**Table 1:** Baseline Participant Characteristics by Infection‐Related Hospitalization Status, Mean±SD or %(N) Among N=14 468 ARIC Participants, 1987 to1989

	Unmatched cohort			Matched cohort		
	No infection‐related hospitalization through 2018 (N=7795)	Infection‐related hospitalization through 2018 (N=6673)	*P* value	No infection‐related hospitalization through 2018 (N=1773)	Infection‐related hospitalization through 2018 (N=4735)	*P* value
Demographics						
Age, y	53.4±5.7	54.9±5.8	<0.0001	53.5±5.7	54.6±5.8	<0.0001
Male sex	45.7% (3562)	45.3% (3021)	0.61	45.1% (799)	43.9% (2077)	0.39
Race/center			<0.0001			0.05
White/Minneapolis	28.4% (2217)	22.9% (1528)		27.6% (489)	24.3% (1149)	
White/Washington	23.6% (1840)	27.8% (1855)		24.6% (436)	26.9% (1274)	
White/Forsyth	22.0% (1714)	24.1% (1606)		22.8% (404)	23.9% (1134)	
Black/Forsyth	2.7% (208)	3.2% (215)		3.4% (60)	3.1% (146)	
Black/Mississippi	23.3% (1816)	22.0% (1469)		21.7% (384)	21.8% (1032)	
Education			<0.0001			0.0002
Basic	19.6% (1531)	26.5% (1769)		20.5% (364)	25.0% (1186)	
Intermediate	41.1% (3203)	41.2% (2747)		41.6% (738)	41.3% (1954)	
Advanced	39.3% (3061)	32.3% (2157)		37.9% (671)	33.7% (1595)	
Insurance status (Yes)	91.1% (7104)	89.9% (6002)	0.01	91.2% (1617)	90.0% (4261)	0.14
Behavioral characteristics						
Physical activity	2.5±0.81	2.4±0.78	<0.0001	2.5±0.80	2.4±0.78	0.002
Smoking status			<0.0001			<0.0001
Never	44.6% (3480)	38.7% (2581)		45.7% (811)	39.7% (1881)	
Former	32.2% (2511)	32.2% (2149)		32.9% (584)	31.6% (1497)	
Current	23.1% (1804)	29.1% (1943)		21.3% (378)	28.7% (1357)	
Clinical characteristics						
Body mass index, kg/m^2^	27.1±5.0	28.0±5.5	<0.0001	27.2±5.2	27.7±5.4	0.001
Systolic blood pressure, mm Hg	119.9±18.5	122.0±18.9	<0.0001	119.4±18.1	121.3±18.9	0.0004
Diastolic blood pressure, mm Hg	73.5±11.1	73.6±11.4	0.30	73.1±10.4	73.6±11.4	0.12
Using blood pressure medication	24.1% (1878)	30.2% (2013)	<0.0001	25.0% (443)	28.3% (1340)	0.01
Total cholesterol, mg/dL	213.8±40.6	215.3±42.1	0.03	212.5±40.2	214.6±42.0	0.07
High‐density lipoprotein cholesterol, mg/dL	52.9±17.1	51.0±16.8	<0.0001	52.6±16.2	51.5±16.9	0.02
Low‐density lipoprotein cholesterol, mg/dL	136.9±38.7	138.6±39.8	0.01	136.0±38.3	137.6±39.6	0.12
Prevalent diabetes	8.3% (647)	13.0% (865)	<0.0001	8.1% (143)	11.6% (547)	<0.0001
Prevalent coronary heart disease	3.6% (283)	4.5% (298)	0.01	3.8% (68)	3.7% (176)	0.82
Prevalent atrial fibrillation	0.1% (7)	0.2% (15)	0.04	0.1% (2)	0.2% (10)	0.41
Prevalent stroke	1.4% (111)	1.6% (106)	0.42	1.4% (24)	1.5% (71)	0.66
Using beta blockers	8.7% (676)	10.9 (729)	<0.0001	8.8% (156)	9.9% (470)	0.17
Using angiotensin‐converting enzyme inhibitors/angiotensin receptor blockers	2.8% (219)	3.5% (235)	0.01	2.0% (36)	3.5% (165)	0.003
Using diuretics	12.2% (951)	15.8% (1056)	<0.0001	12.1% (214)	14.8% (702)	0.004
C‐reactive protein, mg/L	1.9 [0.9–4.1]	2.5 [1.2–5.2]	<0.0001	1.8 [0.9–4.1]	2.4 [1.2–5.0]	<0.0001
NT‐terminal pro‐B‐type natriuretic peptide, pg/mL	49.1 [26.3–88.7]	53.8 [28.3–98.2]	<0.0001	49.9 [27.8–87.5]	51.0 [27.0–93.8]	0.40

Means±SD and median [25th percentile, 75th percentile] for continuous variables and N (%) for categorical variables. ARIC indicates Atherosclerosis Risk in Communities.

### Infection‐Related Hospitalization and Incident Heart Failure

**Figure 2:**
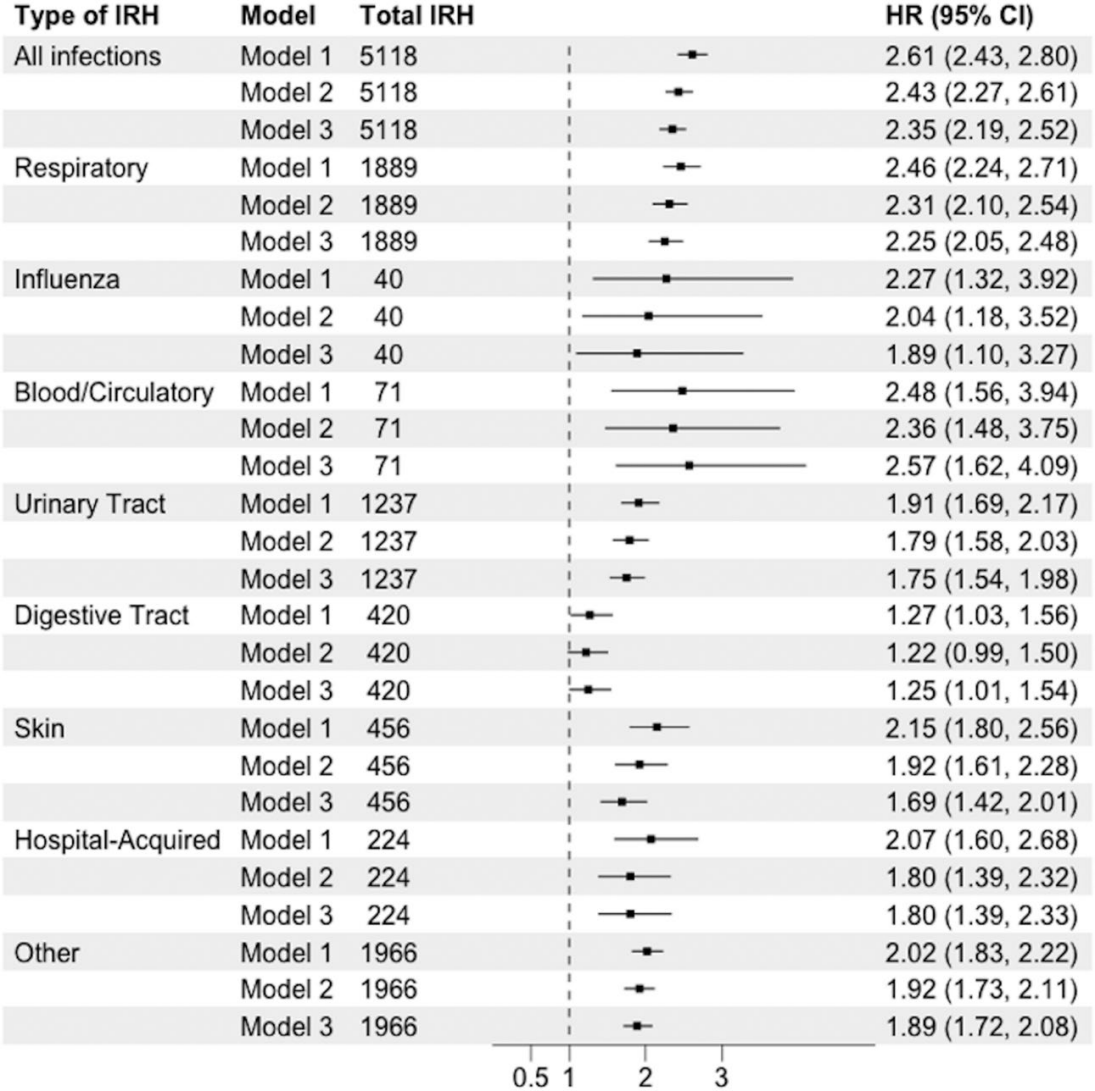
Multivariable adjusted hazard ratios (95% CI) for the association between infection‐related hospitalization in the first 5 positions and incident heart failure among N=14 468 ARIC participants 1987 to 2018. Total of 3565 incident HF events. Model 1: adjusted for covariates measured in 1987 to 1989: age, sex, race/center education, health insurance. Model 2: model 1+physical activity, smoking status, body mass index. Model 3: model 2+diabetes, systolic blood pressure, antihypertensive medication use, low‐density lipoprotein cholesterol, prevalent coronary heart disease. ARIC indicates Atherosclerosis Risk in Communities; HR, hazard ratio; and IRH, infection‐related hospitalization.

Between visit 1 (1987–1989) and 2018, 3565 (25%) had incident HF, and the incidence rate was 107.6 events per 10 000 person‐years. The mean time between IRH and HF was 7±6 years and 82% of incident HF cases developed >1 year following IRH. IRH was associated with any incident HF after multivariable adjustment (Figure 2). Results for the matched analysis were slightly attenuated compared with our unmatched results, with an HR of 1.62 (95% CI, 1.45–1.82) in the fully adjusted model, model 2 (Table 2). Results from the unmatched Cox model indicate that the rate of HF among those with an IRH was 2.35 (95% CI, 2.19–2.52) compared with those who did not have an IRH in the fully adjusted model (model 3). This relationship was generally consistent across different types of infections (Figure 2). Results were not meaningfully changed in sensitivity analyses that (1) removed any incident HF event occurring within 1 year of infection to minimize the potential for reverse causality (Table S2); or (2) included additional adjustments for cardiovascular disease medications, C‐reactive protein, or NT‐proBNP (N‐ terminal pro‐B‐type natriuretic peptide; Table S3).

**Table 2:** Matched Analyses With Multivariable Adjusted Hazard Ratios (95% CI) of the Association Between Infection‐Related Hospitalization in the First 5 Positions and Incident Heart Failure Among ARIC Participants 1987 to 2018

	Total sample	No. matched uninfected that became infected	No. infections	No. incident HF	HR (95% CI)	*P* value
Model 1	6508	1481	4735	1556	1.68 (1.50–1.88)	<0.0001
Model 2	6508	1481	4735	1556	1.62 (1.45–1.82)	<0.0001

Model 1: Unadjusted (but matched for age, sex, race/center, and diabetes status at baseline). Model 2: model 1+ adjusted for covariates measured in 1987 to 1989: education, insurance, body mass index, smoking status, low‐density lipoprotein cholesterol, physical activity, hypertension medication, prevalent coronary heart disease, and systolic blood pressure. ARIC indicates Atherosclerosis Risk in Communities; HF, heart failure; and HR, hazard ratio.

In sensitivity analyses restricting follow‐up time at the 25th, 50th, and 75th percentile of the follow‐up time distribution, the HRs for incident HF comparing participants with versus without infection were as follows: (1) 2.5 years of follow‐up (25th percentile) HR=2.13 (95% CI, 1.72–2.64); (2) 6 years of follow‐up (50th percentile) HR=1.77 (95% CI, 1.52–2.05); and (3) 12.3 years of follow‐up (75th percentile of follow‐up time) HR=1.62 (95% CI, 1.43–1.83). Although the relationship attenuates as follow‐up time increases, the results remained statistically and clinically significant.

**Figure 3:**
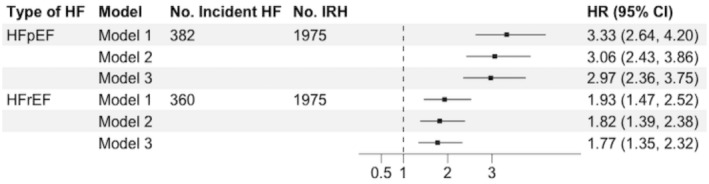
Multivariable adjusted hazard ratios (95% CI) for the association between all infection‐related hospitalizations in the first 5 positions and incident HFrEF and HFpEF among N=7669 ARIC participants 1996 to 2018. Total of 1975 infections. Model 1: adjusted for covariates measured in 1996 to 1998: age, sex, race/center education, health insurance. Model 2: model 1+physical activity, smoking status, body mass index. Model 3: model 2+diabetes, systolic blood pressure, antihypertensive medication use, low‐density lipoprotein cholesterol, prevalent coronary heart disease. ARIC indicates Atherosclerosis Risk in Communities; HFpEF, heart failure with preserved ejection fraction; HFrEF, heart failure with reduced ejection fraction; HR, hazard ratio; and IRH, infection‐related hospitalization.

Among 7669 HF participants in 2005 for whom HF subtype adjudication was available, median follow‐up time was 13 years. The cumulative incidence of the HF subtypes between 2005 and 2018 was 4.7% (HFrEF, 360/7669) and 5.0% (HFpEF, 382/7669), respectively. The incidence rates of HFrEF and HFpEF were 42.2 per 10 000 person‐years and 44.8 per 10 000‐person years, respectively. After multivariable adjustment, IRH was associated with both HFrEF and HFpEF (HR, 1.77 [95% CI, 1.35–2.32] and 2.97 [95% CI, 2.36–3.75]), respectively (Figure 3).

### Sensitivity Analyses

Results remained statically significant, though slightly attenuated when restricting our exposure definition for IRH to *ICD‐9/10* codes in the first position of diagnosis discharge compared with anywhere in the first 5 positions (Table 3). This relationship persisted across different infection types (respiratory, influenza, urinary tract, digestive tract, skin, blood/circulatory system, hospital‐acquired, and other infections) (Figures 2 and 3). In the fully adjusted model, model 3, blood/circulatory and respiratory infections had the strongest associations with HF (HR, 2.57 [95% CI, 1.62–4.09] and 2.25 [95% CI, 2.05–2.48]), respectively. In contrast, digestive tract infections were only marginally associated with HF (HR, 1.25 [95% CI, 1.01–1.54]).

**Table 3:** Multivariable Adjusted Hazard Ratios (95% CI) of the Association Between Infection‐Related Hospitalization in the First Position and Incident Heart Failure Among N=14 468 ARIC Participants 1987 to 2018

	No. infections	HR (95% CI)	*P* value
All infections			
Model 1	3092	2.52 (2.33–2.74)	<0.0001
Model 2	3092	2.34 (2.16–2.54)	<0.0001
Model 3	3092	2.28 (2.10–2.47)	<0.0001
Influenza			
Model 1	35	2.40 (1.33–4.34)	0.004
Model 2	35	1.90 (1.05–3.44)	0.03
Model 3	35	1.82 (1.01–3.30)	0.05
Respiratory infections			
Model 1	1096	2.62 (2.34–2.94)	<0.0001
Model 2	1096	2.44 (2.17–2.74)	<0.0001
Model 3	1096	2.40 (2.14–2.69)	<0.0001
Blood/circulatory system infection			
Model 1	26	4.73 (2.36–9.46)	<0.0001
Model 2	26	4.80 (2.40–9.61)	<0.0001
Model 3	26	3.83 (1.91–7.68)	0.0002
Urinary tract infection			
Model 1	333	1.45 (1.13–1.87)	0.003
Model 2	333	1.42 (1.10–1.82)	0.01
Model 3	333	1.34 (1.04–1.72)	0.02
Digestive tract infection			
Model 1	269	1.16 (0.89–1.50)	0.27
Model 2	269	1.12 (0.87–1.45)	0.39
Model 3	269	1.11 (0.85–1.43)	0.45
Skin infection			
Model 1	271	2.52 (2.04–3.11)	<0.0001
Model 2	271	2.22 (1.80–2.75)	<0.0001
Model 3	271	2.04 (1.65–2.52)	<0.0001
Hospital‐acquired infection			
Model 1	193	2.63 (2.03–3.42)	<0.0001
Model 2	193	2.28 (1.75–2.96)	<0.0001
Model 3	193	2.14 (1.65–2.78)	<0.0001
Other infections			
Model 1	905	2.34 (2.02–2.70)	<0.0001
Model 2	905	2.21 (1.91–2.56)	<0.0001
Model 3	905	2.26 (1.95–2.61)	<0.0001

Total of 3560 incident heart failure events. Model 1: adjusted for covariates measured in 1987 to 1989: age, sex, race/center education, health insurance. Model 2: model 1+physical activity, smoking status, body mass index. Model 3: model 2+diabetes, systolic blood pressure, antihypertensive medication use, low‐density lipoprotein cholesterol, prevalent coronary heart disease. ARIC indicates Atherosclerosis Risk in Communities; and HR, hazard ratio.

## DISCUSSION

We found IRH to be associated with incident HF during up to 31 years of follow‐up. Findings were consistent across several, but not all, infection types and when considering both HFrEF and HFpEF, with results being empirically stronger for incident HFpEF and during the first 2.5 years of follow‐up. The observed associations remained consistent after extensive adjustment for sociodemographic, behavioral, and HF risk biomarkers along with other comorbidities.

Few studies have evaluated infection as a risk factor for new onset HF^23^, ^24^ though some prior studies have assessed infection and incident cardiovascular events.^30^, ^33^, ^34^, ^35^ A previous report from ARIC observed that in‐ and outpatient infections increased the risk of coronary artery disease and ischemic stroke.^30^, ^36^ Studies investigating HF outcomes following infection are less common, and most have focused specifically on pneumonia. Eurich et al. evaluated the risk of HF after community‐acquired pneumonia during 10 years of follow‐up^37^ and reported that community‐acquired pneumonia increased the risk of HF. Similarly, 3 additional studies have reported elevated incidence of HF within 30 days of community‐acquired pneumonia, with reported rates ranging from 1.4% in outpatient populations^33^ to as high as 24% among inpatients.^23^, ^38^ In ARIC, pneumonia was included within the respiratory infection category and therefore not assessed as a separate exposure. Similarly, prior studies have found COVID‐19 hospitalization to be associated with increased risk of incident HF.^39^, ^40^

Our findings that infection‐related hospitalizations are associated with incident HF have a plausible biological rationale. Chronic nonresolving inflammation secondary to severe infections is one possible pathophysiological mechanism linking infection to HF. A normal inflammatory response prompted by an infection is characterized by the temporally restricted upregulation of inflammatory activity that occurs when an infection is present, which then resolves once the threat has passed.^41^, ^42^ However, biological, psychological, environmental, and social factors may delay or prevent resolution of this acute phase and result in chronic inflammation and immune activation.^43^ Shifts in the inflammatory response from short to long lived can cause a breakdown of immune tolerance,^41^, ^44^ leading to major alterations in end‐organ structure and function.^41^, ^44^, ^45^, ^46^, ^47^, ^48^ Notable examples of low‐grade asymptomatic inflammation causing end‐organ damage include obesity^49^ and aging, as evidenced by an increase in circulating levels of TNF‐α (tumor necrosis factor alpha) and IL‐6 (interleukin‐6).^50^

As inflammation is implicated in both HFpEF and HFrEF, but is potentially more prominent in HFpEF, this might explain why our findings were stronger for HFpEF versus HFrEF. Inflammatory cytokines, such as IL‐6 and TNF‐α, exert direct effects on myocardial and vascular cells that predispose individuals to HF,^51^, ^52^ and these biomarkers are elevated in patients with HfrEF and HfpEF, although stronger associations have been reported in the context of HfpEF.^53^ This was demonstrated in 2 recent analyses from COACH (Counseling in Heart Failure) and BIOSTAT‐CHF (Biology Study to Tailored Treatment in Chronic Heart Failure), which found a stronger relationship between biomarkers of inflammation and HfpEF compared with HfrEF.^54^, ^55^ It is also possible that the increased burden of proinflammatory comorbidities in HfpEF, such as diabetes, hypertension, chronic obstructive pulmonary disease, obesity, and chronic kidney disease, may account for these findings.^56^, ^57^

Infection and HF also have shared risk factors, such as diabetes,^58^, ^59^, ^60^ stroke,^61^, ^62^ and older age.^63^, ^64^ One of the most frequent causes of HF is ischemic heart disease, which leads to the loss of myocardial tissue and contractile force.^65^ Patients with ischemic heart disease who develop HF have a clinical history of myocardial infarction with atherosclerotic disease of epicardial arteries.^66^, ^67^ Similarly, acute infections are known to be associated with an increased risk of myocardial infarction; in particular, respiratory tract infections, including pneumonia, bronchitis, and influenza, in addition to digestive and urinary tract infections, have been associated.^68^ Our results suggest that IRH may contribute and ultimately lead to HF. Future studies are necessary to validate causal association between infections and HF development.

### Strengths and Limitations

Some important limitations to this study should be noted. We used covariates that were measured once at baseline (visit 1 for our analysis examining any HF and visit 4 for our analysis examining HF subtypes) to estimate remote associations for the outcomes that occurred over the next 31 years. In addition, although we adjusted for several covariables in order to reduce confounding, given that this is an observational cohort study, unmeasured confounding may still be present. This is particularly important in the case of potentially undiagnosed chronic obstructive pulmonary disease, which could have confounded our findings. Similarly, undiagnosed or unreported autoimmune diseases could have confounded our results. Differential loss to follow‐up related to risk for infection (or incident HF) could have contributed to selection bias. For example, very sick participants could die before developing HF (or clinically apparent HF) because HF can progress slowly. There is also some potential for reverse causality. It is possible that asymptomatic or undiagnosed HF was present at baseline and could have contributed to future infection‐related hospitalization, giving the spurious appearance of increased HF incidence among people with IRH.

In addition, the use of *ICD* codes can lead to misclassification, though a prior validation of HF hospitalizations indicated that the positive predictive value of 428.x was 93% for acute decompensated HF and 97% for chronic HF.^28^ ARIC implemented a rigorous approach to adjudicate HFrEF and HFpEF, and prior studies also used the first 5 *ICD* positions when assessing infection in ARIC.^30^ Our analysis did not consider less severe acute or chronic outpatient infections (ie those infections that did not require inpatient hospitalization), which are also hypothesized to be a risk factor for incident HF. For example, our prior work reported a relationship between periodontal infections and incident HFpEF and HFrEF.^69^ The lack of information on complete infectious history is likely to be nondifferential and would bias results toward the null in expectation.

There were also many strengths to this study, including the study population composed of a large, multiracial, community‐based cohort of participants followed for up to 31 years (from 1987–1989 to 2018). In addition, the rigorous approach used to adjudicate HFrEF and HFpEF in ARIC enabled focused analyses for HF subtypes in the same study, which has not been performed previously. Our exposure assessment was time varying, allowing for risk to begin accruing immediately following IRH as opposed to using a participant self‐report of historical IRH without knowledge of IRH timing. We were further able to assess specific categories of infections in relation to our outcome as opposed to any (ie uncategorized) infection.

## CONCLUSIONS

We have observed IRH to be associated with incident HF among a diverse, community‐based sample of adults. Findings were notably stronger among those with HFpEF, for which treatment options are limited. Our findings support prior literature linking infection to HF risk as well as the need for more research exploring the potential for infection‐prevention strategies, such as vaccination, to minimize HF burden. Moreover, history of infection could potentially become an important tool for risk assessment and patient management. If future studies were to provide evidence of causal association, there could be significant population‐level implications given the high prevalence of infections and the burden of HF on our aging society.

## Sources of Funding

The Atherosclerosis Risk in Communities study has been funded in whole or in part with Federal funds from the National Heart, Lung, and Blood Institute, National Institutes of Health, Department of Health and Human Services, under Contract nos. (HHSN268201700001I, HHSN268201700002I, HHSN268201700003I, HHSN268201700004I, HHSN268201700005I). Rebecca Molinsky was supported by institutional training grant T32HL007779 from the National Institutes of Health.

## Disclosures

None.

## Supporting information

Tables S1–S3
