# Supplementary material for: Infection‐Related Hospitalization and Incident Heart Failure: The Atherosclerosis Risk in Communities Study
Source: J Am Heart Assoc. 2025 Jan 30;14(3):e033877. doi: 10.1161/JAHA.123.033877 (PMC12074748; doi:10.1161/JAHA.123.033877)
Supplement: Supplementary file 1 — Tables S1–S3 [file JAH3-14-e033877-s001.pdf]

Supplemental Material

Table S1. **International Classification of Diseases (ICD) Codes Utilized for the Ascertainment of Heart Failure.**

**Description**	**ICD-9-CM Code**	**ICD-10-CM Code**
Heart failure	428.XX	I50
Congestive heart failure, unspecified	428.0	I50.814, I50.9
Left heart failure	428.1	I50.1
Systolic heart failure	428.2	
Systolic heart failure, unspecified	428.20	I50.20
Acute systolic heart failure	428.21	I50.21
Chronic systolic heart failure	428.22	I50.22
Acute on chronic systolic heat failure	428.23	I50.23
Diastolic heart failure	428.3	
Diastolic heart failure, unspecified	428.30	I50.30
Acute diastolic heart failure	428.31	I50.31
Chronic diastolic heart failure	428.32	50.32
Acute on chronic diastolic heart failure	428.33	I50.33
Combined systolic and diastolic heart failure	428.4	
Combined systolic and diastolic heart failure, unspecified	428.40	I50.40
Acute combined systolic and diastolic heart failure	428.41	I50.41
Chronic combined systolic and diastolic heart failure	428.42	I50.42
Acute on chronic combined systolic and diastolic heart failure	428.43	I50.43
Heart failure, unspecified	428.9	I50.810, I50.811, I50.812, I50.813, I50.82, I50.83, I50.84, I50.89, I50.9
Rheumatic heart failure (congestive)	398.91	I09.81
Malignant hypertensive heart disease with heart failure	402.01	I11.0
Benign hypertensive heart disease with heart failure	402.11	
Unspecified hypertensive heart disease with heart failure	402.91	
Malignant hypertensive heart and renal disease with heart failure	404.01	I13.0
Benign hypertensive heart and renal disease with heart failure	404.11	

Unspecified hypertensive heart and renal disease with heart failure	404.91	
Malignant hypertensive heart and renal disease with heart failure and renal failure	404.03	I13.2
Benign hypertensive heart and renal disease with heart failure and renal failure	404.13	
Unspecified hypertensive heart and renal disease with heart failure and renal failure	404.93	
ICD-9- [10]-CM = International Classification of Diseases, Ninth [Tenth] Revision, Clinical Modification		

Table S2. Multivariable adjusted hazard ratios (95% confidence interval) of the association between infection related hospitalization in the first five positions and incident heart failure occurring greater than 1 year after infection among N=14,229 ARIC participants 1987-2018

	# Infections	HR (95%CI)	p-value
**All Infections**			
Model 1	4,879	2.07 (1.92, 2.24)	<.0001
Model 2	4,879	1.93 (2.16, 2.54)	<.0001
Model 3	4,879	1.86 (1.72, 2.00)	<.0001
**Influenza**			
Model 1	37	1.86 (1.00, 3.47)	0.05
Model 2	37	1.67 (0.90, 3.11)	0.10
Model 3	37	1.55 (0.83, 2.88)	0.17
**Respiratory Infections**			
Model 1	1,774	1.97 (1.77, 2.19)	<.0001
Model 2	1,774	1.85 (1.66, 2.05)	<.0001
Model 3	1,774	1.80 (1.62, 2.00)	<.0001
**Blood/Circulatory System Infection**			
Model 1	68	2.21 (1.33, 3.67)	0.002
Model 2	68	2.10 (1.26, 3.49)	0.004

Model 3	68	2.28 (1.37, 3.79)	0.002
**Urinary Tract Infection**			
Model 1	1176	1.56 (1.35, 1.79)	<.0001
Model 2	1176	1.45 (1.26, 1.67)	<.0001
Model 3	1176	1.42 (1.23, 1.63)	<.0001
**Digestive Tract Infection**			
Model 1	407	1.15 (0.92, 1.45)	0.22
Model 2	407	1.11 (0.88, 1.39)	0.39
Model 3	407	1.13 (0.90, 1.42)	0.30
**Skin Infection**			
Model 1	434	1.90 (1.57, 2.30)	<.0001
Model 2	434	1.69 (1.39, 2.04)	<.0001
Model 3	434	1.48 (1.22, 1.79)	<.0001
**Hospital-Acquired Infection**			
Model 1	214	1.82 (1.37, 2.42)	<.0001
Model 2	214	1.57 (1.18, 2.09)	0.002
Model 3	214	1.58 (1.19, 2.10)	0.002
**Other Infections**			
Model 1	1889	1.77 (1.60, 1.97)	<.0001
Model 2	1889	1.67 (1.50, 1.86)	<.0001

Model 3	1889	1.65 (1.49, 1.84)	<.0001

Total of 3,326 incident HF events
Model 1: adjusted for covariates measured in 1987-89: age, sex, race/center education, health insurance
Model 2: model 1 + physical activity, smoking status, BMI
Model 3: model 2 + diabetes, systolic blood pressure, antihypertensive medication use, LDL cholesterol, prevalent CHD

Table S3. Multivariable adjusted hazard ratios (95% confidence interval) of the association between infection related hospitalization in the first position and incident heart failure with adjustments for cardiovascular medications, C-reactive protein, and NT-proBNP among N=14,463 ARIC participants 1987-2018

		N	Total IRH	HF	HR (95% CI)	p-value
All infections	Model 1	14,463	3,029	3,560	2.52 (2.32, 2.73)	<.0001
	Model 2	14,463	3,029	3,560	2.34 (2.16, 2.54)	<.0001
	Model 3	14,463	3,029	3,560	2.27 (2.10, 2.47)	<.0001
	Model 4a	14,463	3,029	3,560	2.28 (2.10, 2.48)	<.0001
	Model 4b	14,463	3,029	3,560	2.29 (2.11, 2.49)	<.0001
	Model 4c	14,463	3,029	3,560	2.27 (2.09, 2.47)	<.0001
	Model 4d	14,463	3,029	3,560	2.30 (2.12, 2.49)	<.0001
	Model 5a	12,041	2,418	2,802	2.18 (1.98, 2.40)	<.0001
	Model 5b	12,041	2,418	2,802	2.20 (2.00, 2.41)	<.0001
	Model 5c	12,041	2,418	2,802	2.15 (1.96, 2.37)	<.0001
	Model 5d	12,041	2,418	2,802	2.10 (1.91, 2.31)	<.0001
Respiratory	Model 1	14,463	1,096	3,560	2.62 (2.34, 2.94)	<.0001
	Model 2	14,463	1,096	3,560	2.44 (2.17, 2.74)	<.0001
	Model 3	14,463	1,096	3,560	2.40 (2.14, 2.69)	<.0001
	Model 4a	14,463	1,096	3,560	2.40 (2.14, 2.69)	<.0001
	Model 4b	14,463	1,096	3,560	2.39 (2.13, 2.68)	<.0001
	Model 4c	14,463	1,096	3,560	2.40 (2.14, 2.69)	<.0001
	Model 4d	14,463	1,096	3,560	2.40 (2.14, 2.70)	<.0001
	Model 5a	12,041	878	2,802	2.33 (2.04, 2.66)	<.0001
	Model 5b	12,041	878	2,802	2.39 (2.09, 2.73)	<.0001
	Model 5c	12,041	878	2,802	2.29 (2.00, 2.62)	<.0001
	Model 5d	12,041	878	2,802	2.28 (2.00, 2.61)	<.0001
Influenza	Model 1	14,463	35	3,560	2.40 (1.33, 4.34)	0.004
	Model 2	14,463	35	3,560	1.90 (1.05, 3.44)	0.03

	Model 3	14,463	35	3,560	1.82 (1.01, 3.30)	0.05
	Model 4a	14,463	35	3,560	1.82 (1.00, 3.29)	0.05
	Model 4b	14,463	35	3,560	1.77 (0.98, 3.21)	0.06
	Model 4c	14,463	35	3,560	1.76 (0.97, 3.18)	0.06
	Model 4d	14,463	35	3,560	1.78 (0.98, 3.23)	0.06
	Model 5a	12,041	30	2,802	2.06 (1.11, 3.85)	0.02
	Model 5b	12,041	30	2,802	2.40 (1.28, 4.47)	0.01
	Model 5c	12,041	30	2,802	1.83 (0.98, 3.42)	0.06
	Model 5d	12,041	30	2,802	2.25 (1.21, 4.19)	0.01
Blood/Circulatory	Model 1	14,463	26	3,560	4.73 (2.36, 9.46)	<.0001
	Model 2	14,463	26	3,560	4.80 (2.40, 9.60)	<.0001
	Model 3	14,463	26	3,560	3.83 (1.91, 7.68)	0.0002
	Model 4a	14,463	26	3,560	4.14 (2.06, 8.29)	<.0001
	Model 4b	14,463	26	3,560	4.04 (2.02, 8.10)	<.0001
	Model 4c	14,463	26	3,560	3.91 (1.95, 7.84)	0.0001
	Model 4d	14,463	26	3,560	3.76 (1.87, 7.54)	0.0002
	Model 5a	12,041	20	2,802	4.14 (1.85, 9.25)	0.001
	Model 5b	12,041	20	2,802	3.30 (1.48, 7.38)	0.004
	Model 5c	12,041	20	2,802	3.29 (1.47, 7.33)	0.004
	Model 5d	12,041	20	2,802	3.15 (1.41, 7.06)	0.01
Urinary Tract	Model 1	14,463	333	3,560	1.45 (1.13, 1.87)	0.003
	Model 2	14,463	333	3,560	1.41 (1.10, 1.82)	0.01
	Model 3	14,463	333	3,560	1.34 (1.04, 1.72)	0.02
	Model 4a	14,463	333	3,560	1.34 (1.04, 1.72)	0.02
	Model 4b	14,463	333	3,560	1.37 (1.07, 1.76)	0.01
	Model 4c	14,463	333	3,560	1.31 (1.02, 1.68)	0.04
	Model 4d	14,463	333	3,560	1.35 (1.05, 1.73)	0.02
	Model 5a	12,041	279	2,802	1.22 (0.90, 1.64)	0.20
	Model 5b	12,041	279	2,802	1.27 (0.94, 1.71)	0.12
	Model 5c	12,041	279	2,802	1.34 (0.99, 1.81)	0.05
	Model 5d	12,041	279	2,802	1.25 (0.93, 1.68)	0.15

Digestive Tract	Model 1	14,463	269	3,560	1.16 (0.89, 1.50)	0.27
	Model 2	14,463	269	3,560	1.12 (0.87, 1.45)	0.39
	Model 3	14,463	269	3,560	1.11 (0.85, 1.43)	0.45
	Model 4a	14,463	269	3,560	1.13 (0.87, 1.46)	0.36
	Model 4b	14,463	269	3,560	1.13 (0.87, 1.47)	0.35
	Model 4c	14,463	269	3,560	1.10 (0.85, 1.43)	0.46
	Model 4d	14,463	269	3,560	1.14 (0.88, 1.47)	0.33
	Model 5a	12,041	211	2,802	1.00 (0.73, 1.37)	0.99
	Model 5b	12,041	211	2,802	1.02 (0.74, 1.39)	0.92
	Model 5c	12,041	211	2,802	0.94 (0.69, 1.29)	0.72
	Model 5d	12,041	211	2,802	0.99 (0.72, 1.36)	0.95
Skin	Model 1	14,463	271	3,560	2.51 (2.04, 3.11)	<.0001
	Model 2	14,463	271	3,560	2.22 (1.80, 2.75)	<.0001
	Model 3	14,463	271	3,560	2.04 (1.65, 2.52)	<.0001
	Model 4a	14,463	271	3,560	2.03 (1.64, 2.51)	<.0001
	Model 4b	14,463	271	3,560	2.03 (1.64, 2.51)	<.0001
	Model 4c	14,463	271	3,560	2.04 (1.65, 2.52)	<.0001
	Model 4d	14,463	271	3,560	2.05 (1.66, 2.53)	<.0001
	Model 5a	12,041	200	2,802	2.04 (1.59, 2.63)	<.0001
	Model 5b	12,041	200	2,802	2.07 (1.61, 2.66)	<.0001
	Model 5c	12,041	200	2,802	1.98 (1.53, 2.54)	<.0001
	Model 5d	12,041	200	2,802	2.02 (1.57, 2.59)	<.0001
Hospital-Acquired	Model 1	14,463	193	3,560	2.63 (2.03, 3.42)	<.0001
	Model 2	14,463	193	3,560	2.28 (1.75, 2.96)	<.0001
	Model 3	14,463	193	3,560	2.14 (1.65, 2.78)	<.0001
	Model 4a	14,463	193	3,560	2.20 (1.69, 2.86)	<.0001
	Model 4b	14,463	193	3,560	2.17 (1.67, 2.82)	<.0001
	Model 4c	14,463	193	3,560	2.15 (1.65, 2.79)	<.0001
	Model 4d	14,463	193	3,560	2.19 (1.69, 2.85)	<.0001
	Model 5a	12,041	156	2,802	1.80 (1.32, 2.45)	0.0002

	Model 5b	12,041	156	2,802	1.67 (1.22, 2.28)	0.001
	Model 5c	12,041	156	2,802	1.76 (1.29, 2.40)	0.0003
	Model 5d	12,041	156	2,802	1.45 (1.06, 1.97)	0.02
Other	Model 1	14,463	905	3,560	2.34 (2.02, 2.70)	<.0001
	Model 2	14,463	905	3,560	2.21 (1.91, 2.55)	<.0001
	Model 3	14,463	905	3,560	2.25 (1.95, 2.61)	<.0001
	Model 4a	14,463	905	3,560	2.24 (1.94, 2.59)	<.0001
	Model 4b	14,463	905	3,560	2.26 (1.95, 2.61)	<.0001
	Model 4c	14,463	905	3,560	2.27 (1.96, 2.62)	<.0001
	Model 4d	14,463	905	3,560	2.26 (1.96, 2.62)	<.0001
	Model 5a	12,041	734	2,802	2.36 (2.00, 2.79)	<.0001
	Model 5b	12,041	734	2,802	2.36 (2.00, 2.78)	<.0001
	Model 5c	12,041	734	2,802	2.32 (1.97, 2.74)	<.0001
	Model 5d	12,041	734	2,802	2.33 (1.97, 2.75)	<.0001

Model 1: Adjusted for age, sex, race/center, education, health insurance
Model 2: Model 1 + adjusted for physical activity, smoking status, BMI
Model 3: Model 2 + adjusted diabetes, systolic blood pressure, antihypertensive medication use, LDL cholesterol, prevalent CHD
Model 4a: Model 2 + adjusted diabetes, systolic blood pressure, LDL cholesterol, prevalent CHD, beta blocker use
Model 4b: Model 2 + adjusted diabetes, systolic blood pressure, LDL cholesterol, prevalent CHD, diuretic use
Model 4c: Model 2 + adjusted diabetes, systolic blood pressure, LDL cholesterol, prevalent CHD, ACE inhibitor/ARB use
Model 4d: Model 2 + adjusted diabetes, systolic blood pressure, LDL cholesterol, prevalent CHD, beta blocker use, diuretic use, ACE inhibitor/ARB use
Model 5a: Visit 2 as a baseline (excluding HF and infection events by visit 2), Model 3 + log(C-reactive protein) from visit 2
Model 5b: Visit 2 as a baseline (excluding HF and infection events by visit 2), Model 3 + log(NT-proBNP) from visit 2
Model 5c: Visit 2 as a baseline (excluding HF and infection events by visit 2), Model 3 + log(troponin-T) from visit 2

Model 5d: Visit 2 as a baseline (excluding HF and infection events by visit 2), Model 3 + log(C-reactive protein), log(NT-proBNP) & log(troponin-T) from visit 2
